# Evaluation of Assays for Measurement of Serum (Anti)oxidants in Hemodialysis Patients

**DOI:** 10.1155/2014/843157

**Published:** 2014-05-19

**Authors:** Tatjana Ruskovska, Eugene H. J. M. Jansen, Risto Antarorov

**Affiliations:** ^1^Faculty of Medical Sciences, Goce Delcev University, 2000 Stip, Macedonia; ^2^National Institute for Public Health and the Environment, 3721 MA, Bilthoven, The Netherlands; ^3^General City Hospital, 1000 Skopje, Macedonia

## Abstract

*Background*. Various biomarkers and assays have been used for assessment of (anti)oxidant status in hemodialysis patients, including those intended for measurement of serum total (anti)oxidants, most often as a part of panel biomarkers. *Methods*. Serum (anti)oxidant status was measured in 32 chronically hemodialyzed patients and in 47 healthy persons, using two oxidations and three antioxidant assays. *Results*. The patients before the hemodialysis session have had higher values of total oxidants in comparison to the healthy persons, with a further increase during the hemodialysis. These findings were confirmed with both oxidation assays, but they differ in the percentage of increase and the statistical significance. All three antioxidant assays showed significantly higher values of the total serum antioxidants in the patients before the hemodialysis session in comparison to the healthy persons, and their significant decrease during the hemodialysis. However, the assays differ in the percentage of decrease, its statistical significance, and the correlations with uric acid. *Conclusion*. The variability of results of total (anti)oxidants which are obtained using different assays should be taken into account when interpreting data from clinical studies of oxidative stress, especially in complex pathologies such as chronic hemodialysis.

## 1. Introduction

Oxidative stress, as a state of substantial prooxidant imbalance between oxidants and antioxidants within the cells and tissues, leads to an oxidative damage of proteins, lipids, and DNA. As such, the oxidative stress implies numerous noncommunicable diseases, including end-stage renal disease [1]. Accumulation of uremic toxins [2], bioincompatibility of the dialyzer's membrane [3], depletion of low molecular antioxidants, in the first place ascorbic acid, during the hemodialysis session [4], and excessive and indiscriminate use of intravenous iron preparations [5] have been described as the main factors which cause oxidative stress in the chronically hemodialyzed patients. There is a large body of evidence that the prooxidant state of the chronically hemodialyzed patients is associated with cardiovascular disease [6–8], renal anemia [9, 10], and mineral and bone disorders [11], which largely contribute to their increased morbidity and mortality.

Although the oxidative stress in chronically hemodialyzed patients has been extensively studied, there is no consensus about the use of (anti)oxidant status biomarkers and assays. Thus, different biomarkers and assays and various combinations of them have been used in different clinical studies. Among others, the assays for measurement of the levels of total oxidants [12] and total antioxidants [13] in serum/plasma have been used in many studies. However, using these assays, sometimes different [14, 15] or even opposite findings are reported [16, 17], especially for the total serum antioxidants. These inconsistent data in the literature could be a result of many factors like variations in the process of hemodialysis itself and/or influence of the analytical factors, that is, the methods used.

Therefore, the aim of this study was to conduct a comparative analysis of some of the assays for quantification of the total oxidants and the total antioxidants in serum samples from chronically hemodialyzed patients both before and after the single hemodialysis session and in a group of healthy persons. More specifically, we have evaluated two commercial assays for determining the serum oxidants, as well as two commercial assays and one in-house assay for measurement of total serum antioxidants. We focused primarily on commercial assays because of the less variability in preparation of reagents and standards, which enables generation of more accurate and precise results.

## 2. Materials and Methods

### 2.1. Samples

Serum samples from 32 chronically hemodialyzed patients and 47 healthy persons were analyzed in this study.

The samples from the chronically hemodialyzed patients who were on hemodialysis treatment for more than 1 year and who were treated with a protocol of three hemodialysis sessions a week were obtained from the Department of Hemodialysis at Military Hospital in Skopje. The mean age of the patients was 61 ± 10 years (range 38–78 years). Blood for analysis was drawn immediately before and after the second hemodialysis session within the week in blood collecting tubes (Sarstedt) containing clot activator. Before the hemodialysis blood was drawn after an overnight fasting. The serum was separated with standard centrifugation procedure and immediately divided in portions which were kept tightly closed at −70°C until analysis [18].

The results obtained from the hemodialyzed patients were compared to those from 47 healthy persons (mean age: 25 ± 3 years), candidates for military service. They were physically and mentally healthy, without any chronic or acute disease, as confirmed with their clinical presentation at the day of blood sampling and the results from the standard medical examinations. They did not report use of any antioxidants and supplements. Fasting blood samples, drawn at about 9:00–9:30 am, were obtained during the scheduled standard systematic medical examination. After the centrifugation, the sera were aliquoted in portions which were kept tightly closed at −70°C until analysis.

The Command of the Military Medical Center in Skopje approved this study, as stated in the document N number 07-1756/1 from the Army mail 2990/80, Army of Republic of Macedonia.

### 2.2. Assays

Within this study the following assays have been evaluated: the reactive oxygen metabolites (ROM), test kit d-ROM from Diacron [19] (Grosseto, Italy); the total oxidant status (TOS), test kit from Rel Assay Diagnostics [20] (Gaziantep, Turkey); the ferric reducing ability of plasma (FRAP), an in-house assay; the total antioxidant status (TAS), test kit from Rel Assay Diagnostics; and the biological antioxidant potential (BAP), test kit from Diacron. We have also measured the serum concentrations of uric acid using the method with uricase, with a dedicated test kit, on the autoanalyzer LX20-Pro (Beckman-Coulter).

#### 2.2.1. Oxidation Assays

The d-ROM assay is intended for measurement of the concentration of total hydroperoxides in serum or heparin plasma. The method was first described by Alberti et al. in 2000 [21]. It is based on the following principle.* In vitro*, in an acidic buffered solution (pH = 4.8), the iron ions are released from the serum (plasma) proteins and catalyze the reaction of transformation of hydroperoxides into alkoxyl and peroxyl radicals, which further react with the chromogen N,N-diethyl-p-phenylenediamine. The concentration of the colored complex is directly proportional to the concentration of the hydroperoxides which are present in the sample. The absorbance is measured at 505 nm, and the results are expressed in CARR U. One CARR U corresponds to 0.08 mg/100 mL H_2_O_2_. The characteristics of the assay were evaluated and validated by Verde et al. in 2002 [22], who report that the assay is reliable even in patients with hyposideremic anemia. In our study the assay has been automated on the autoanalyzer LX20-Pro (Beckman-Coulter).

The TOS assay, developed by Erel in 2005 [23], is intended for measurement of the level of oxidant molecules in various biological samples. The assay is based on the principle of oxidation of the ferrous ion-chelator complex to ferric ion with the oxidants which are present in the sample. The ferric ion further forms a colored complex with a chromogen, and the color intensity is directly proportional to the level of total oxidants. The absorbance is measured at 530 nm. The assay is calibrated with hydrogen peroxide and the results are expressed in *μ*mol H_2_O_2_ Eq/L. We have performed this assay in microtiter plates.

#### 2.2.2. Antioxidant Assays

The FRAP, TAS, and BAP assays are intended for direct measurement of the total antioxidant activity of the sample.

The in-house FRAP assay is a modification of the original method of Benzie and Strain [24]. The method is based on the principle of reduction of the ferric-tripyridyltriazine complex to the ferrous form with the antioxidants which are present in the sample. The increase of absorbance is measured at 593 nm, in a kinetic mode. In a modified method we have used an end-point approach with incubation of exactly 8 minutes. The absorbance has been measured at 600 nm on a microplate autoanalyzer ChemWell [25]. A freshly prepared standard solution of FeSO_4_ has been used for calibration of the assay. The results are expressed in *μ*mol/L FeSO_4_.

The TAS assay, developed by Erel in 2004 [26], is based on the principle of reduction of the dark blue-green colored 2,2′-azino-bis(3-ethylbenzothiazoline-6-sulphonic acid) (ABTS) radical to its colorless reduced form with the antioxidants which are present in the sample. The change of the absorbance is measured at 660 nm. The assay is calibrated with a stable antioxidant standard solution, vitamin E analog—Trolox Equivalent. The results are expressed in *μ*mol/L (*μ*mol Trolox Equivalent/L). We have automated this assay on the autoanalyzer LX20-Pro (Beckman-Coulter).

The BAP assay is based on the ability of a colored solution which contains ferric ions bound to a chromogenic substrate (a thiocyanate derived compound) to decolor upon reduction of ferric to ferrous ions. The absorbance is measured at 505 nm, and the results are expressed as *μ*Eq/L (*μ*Eq ferric ions reducing antioxidants/L). Notably, there is no scientific paper which describes validation and verification of the characteristics of this assay. This information is available from the manufacturer of the reagent kit [27]. In our study the assay was automated on the autoanalyzer LX20-Pro (Beckman-Coulter).

All analyses have been run within one analytical series.

### 2.3. Statistical Analysis

The results from measurements are expressed as mean ± standard deviation. The distribution of the data was assessed with Kolmogorov-Smirnov test, using Statistica 7 software.

The data which are normally distributed were processed with Microsoft Excel software. The statistical significance was calculated with Student's *t*-test (paired, two-sample equal variance or two-sample unequal variance, as appropriate).

For the data which are not normally distributed, the Wilcoxon matched pairs test or the Mann-Whitney  *U*  test was used, as appropriate (Statistica 7 software).

The difference between means was considered as statistically significant when it was *P* < 0.05.

The statistical significance of the coefficients of correlation was assessed according to the number of subjects within the group, using a statistical table [28].

## 3. Results

### 3.1. Oxidation Assays

The results from both of the oxidation assays (d-ROM and TOS) show an increase of the concentration of serum oxidants as a result of the single hemodialysis session. However, there is a difference between the assays with regard to the percentage and the statistical significance of the increase. More precisely, we have measured serum ROM concentrations of 428 ± 120 CARR U before and 463 ± 120 CARR U immediately after the hemodialysis session, which is a statistically significant increase for 8.2% (*P* < 0.025). In contrast, as measured with the TOS assay, the percentage of the increase has been much higher (22.4%), but the difference between the values obtained before (2.46 ± 1.86 *μ*mol H_2_O_2_ Eq/L) and after (3.01 ± 2.38 *μ*mol H_2_O_2_ Eq/L) the single hemodialysis session was not statistically significant (*P* > 0.05). Coefficients of correlation between d-ROM and TOS before and after the single hemodialysis session were −0.083 and 0.160, respectively (*P* > 0.05 for both comparisons).

We have also compared the results for serum oxidants of the patients on hemodialysis and the healthy persons and obtained consistent conclusions.

Namely, both ROM (408 ± 92 CARR U) and TOS (2.01 ± 0.81 *μ*mol H_2_O_2_ Eq/L) levels were lower in the healthy persons in comparison to the patients before the hemodialysis session, but the differences were not statistically significant (*P* > 0.05 for both comparisons). However, the increased values of ROM and TOS after the single hemodialysis session were significantly different from those measured in the healthy persons (*P* = 0.023 for ROM; *P* = 0.029 for TOS).

**Figure 1:**
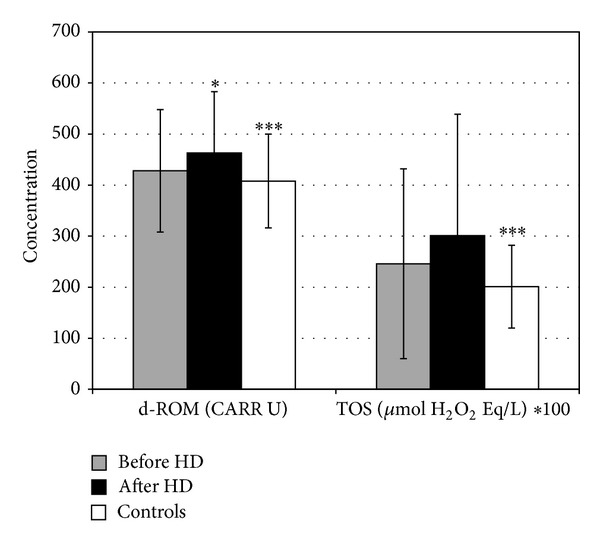
Serum oxidants in patients on chronic hemodialysis and healthy persons. *Statistically significant difference between patients before and after the single hemodialysis session. ***Statistically significant difference between patients after hemodialysis and healthy persons.

The results from the oxidation assays are presented in Figure 1. For better visual presentation the results of the TOS assay are multiplied by 100.

### 3.2. Antioxidant Assays

Before the hemodialysis session, very high values of FRAP in the patients (1758 ± 320 *μ*mol/L), compared to the healthy persons (1378 ± 159 *μ*mol/L), were measured and this difference was highly statistically significant (*P* < 0.001). However, measured immediately after the hemodialysis, the values of FRAP significantly dropped to 1080 ± 162 *μ*mol/L, or on average, for 38.6%, which was again highly statistically significant in comparison to the values before the hemodialysis and to those of the healthy persons (*P* < 0.001 for both comparisons).

Similarly, TAS significantly decreased from 2300 ± 380 *μ*mol/L before the hemodialysis to 1490 ± 240 *μ*mol/L after the hemodialysis session (*P* < 0.001). The percentage of the decrease of TAS (35.2%) was almost identical to that of the FRAP assay. The group of healthy persons have had TAS values of 1550 ± 150 *μ*mol/L, which were significantly lower than those measured in the hemodialyzed patients before the hemodialysis (*P* < 0.001). However, the decline of the TAS values during the hemodialysis session was not so pronounced as to reach a statistical significance in comparison to the healthy persons.

General conclusions which are withdrawn from the BAP assay are the same as those of the FRAP assay. Exceptions are the percentage of the BAP decrease as a result of the single hemodialysis session and the levels of statistical significance of the differences between the healthy subjects and the hemodialysis patients both before and after the single hemodialysis session. More precisely, in the hemodialyzed patients we have measured 2592 ± 239 *μ*Eq/L of BAP before and 2265 ± 268 *μ*Eq/L after the hemodialysis (*P* < 0.001), which is a decrease for only 12.6%. BAP values of the healthy persons were 2442 ± 149 *μ*Eq/L and were significantly different from the values of hemodialyzed patients before and after the hemodialysis (*P* < 0.01 for both comparisons).

**Figure 2:**
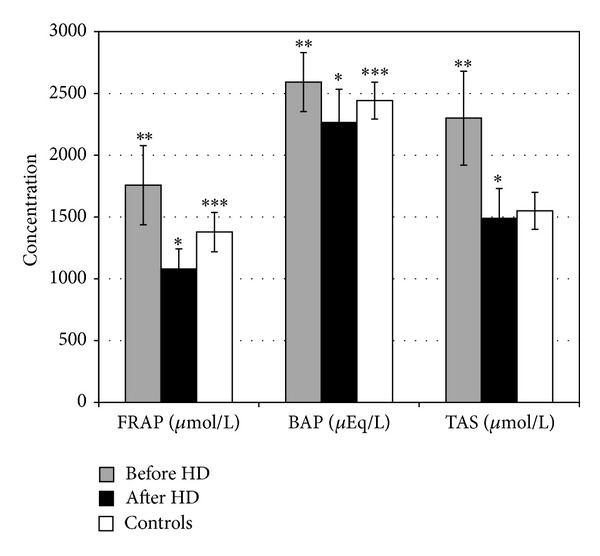
Serum antioxidants in patients on chronic hemodialysis and healthy persons. *Statistically significant difference between patients before and after the single hemodialysis session. **Statistically significant difference between patients before hemodialysis and healthy persons. ***Statistically significant difference between patients after hemodialysis and healthy persons.

The graphic presentation of the results obtained from the antioxidant assays is given in Figure 2. The coefficients of correlation between these assays, both before and after the single hemodialysis session, are given in Table 1.

The serum concentrations of uric acid in the hemodialyzed patients before the hemodialysis session (320 ± 62 *μ*mol/L) were not significantly different from those measured in the healthy persons (303 ± 56 *μ*mol/L), *P* > 0.05. However, during the hemodialysis session, the serum uric acid concentrations decreased to 84 ± 30 *μ*mol/L, which is, on average, a decrease for 73.8%, and which is highly statistically significant in comparison to the values before the hemodialysis and to those of the healthy persons (*P* < 0.001 for both comparisons).

Moreover, the serum uric acid concentrations significantly correlated with FRAP, TAS, and BAP in the hemodialyzed patients, both before and after the hemodialysis session, as well as in the healthy persons (results shown in Table 2).

## 4. Discussion

### 4.1. Oxidation Assays

Two assays, for measurement of the serum oxidants (d-ROM and TOS), were evaluated in this study. These assays are based on completely different principles. Therefore, it was not surprising that the coefficients of correlation between them, both before and after the single hemodialysis session, were not statistically significant. However, the analysis of the results demonstrated that the conclusions which are withdrawn from both assays are generally consistent, yet with some differences.

It has been shown with both of the oxidation assays that before the hemodialysis session the patients had higher concentrations of serum oxidants than the healthy persons, but the difference was not statistically significant.

In other clinical studies, using the d-ROM assay [14, 29], higher values of serum oxidants were also found in hemodialyzed patients in comparison to healthy persons. Notably, in both studies the difference between the patients and the healthy persons was statistically significant, but the *P* values were above 0.025, indicating small differences between the groups. However, in another study [30] the difference of the ROM concentrations between hemodialyzed patients and healthy persons was more pronounced.

It has been also shown with both of the oxidation assays that there was an increase in the concentrations of serum oxidants during the hemodialysis session, leading to a statistically significant difference between the hemodialyzed patients and the healthy subjects.

Others, using the d-ROM assay, have also found a significant increase of ROM levels during the single hemodialysis session [14], but there is also a different finding [15].

To the best of our knowledge, the levels of serum oxidants in hemodialyzed patients have not been measured before with the TOS assay from Rel Assay Diagnostics.

Altogether, although not specific for a particular oxidant or oxidation mechanism and based on completely different principles, the d-ROM and TOS assays in this study consistently reflected the oxidation state of the hemodialyzed patients, which are slightly increased oxidation in comparison to the healthy subjects and further increase during the single hemodialysis session. However, assessing the increase of serum oxidants during the single hemodialysis session, we found an inconsistence between these two assays with regard to the percentage of the increase and its statistical significance, which warrants caution in the interpretation of the results.

### 4.2. Antioxidant Assays

After the introduction of the concept of oxidative stress as an imbalance between the molecules with prooxidant and antioxidant activity, a number of methods for measurement of total nonenzymatic plasma antioxidant activity have been published. There is almost no disease or condition where the total nonenzymatic plasma antioxidant activity has not been assessed. However, the intention to relate* directly* the levels of total antioxidants in food with the total plasma antioxidant activity is not justified because of the known ADME (absorption, distribution, metabolism, and elimination) effects of the human body on the nutritional antioxidants [31]. In addition, it is also not justifiable to relate the values of the total plasma antioxidant activity, directly and in an indiscriminate manner, with the state of good health. The chronically hemodialyzed patients are such an example. Still, the total antioxidant assays remain a useful tool for oxidative stress research, in carefully designed clinical studies, as members of panel biomarkers and always in correlation with other clinical data.

Three photometric assays for direct measurement of the total antioxidant activity (FRAP, TAS, and BAP) have been evaluated in this study, all of them based on different principles. Namely, the FRAP and BAP assays are based on the principle of reduction of ferric to ferrous ions but with use of different complexes, while the TAS assay is based on the principle of reduction of ABTS radical. All three assays are nonspecific and are intended for measurement of various antioxidants which are present in the sample. Relative contributions of the most common plasma antioxidants to the values of FRAP [24] and TAS [26] are given in Table 3. The manufacturer does not provide such data for the BAP assay, but referring to the FRAP assay which also uses the principle of reduction of ferric ions presents data from experiments with uric acid, bilirubin, ascorbic acid, and *α*-tocopherol [27]. Since hyperbilirubinemia is not an issue in the patients included in this study (data not shown) and proteins are nondialyzable molecules, we focus on uric acid and its correlations with the total antioxidant status assays, both before and after a single hemodialysis session.

When the FRAP, TAS, and BAP assays were applied on the same sets of serum samples from hemodialyzed patients and healthy subjects, the results generally reflected the same trends, although there were also some differences. In addition, all three assays correlated significantly with each other both before and after the single hemodialysis session, with exception of the correlation between the FRAP and BAP assays after the hemodialysis session.

Before the hemodialysis session the patients had significantly higher levels of total antioxidants in comparison to the healthy persons, which has been demonstrated with all three assays.

This, at a first glance paradoxical finding, is in accordance with the results from other studies where the total antioxidant status was measured with the FRAP assay [32, 33]. However, there are also some opposite findings [34]. To the best of our knowledge, the BAP assay from Diacron has not been used before for a parallel measurement of total antioxidants in hemodialyzed patients and healthy persons. There is also no information that the TAS assay from Rel Assay Diagnostics had been applied on serum samples from hemodialyzed patients. However, an assay based on the ABTS reduction principle has been used before [35] and the authors report higher levels of total antioxidants in chronically hemodialyzed patients in comparison to healthy persons.

The high levels of total serum antioxidants in the hemodialyzed patients before the single hemodialysis session have been attributed to the abundance of small molecules with reductive properties, mostly the uric acid [36, 37]. However, we did not find that the slightly higher serum uric acid concentrations of the patients before the hemodialysis session were significantly different from those measured in the healthy subjects. As such, it appears that the uric acid is not a primary cause of the remarkably high levels of total antioxidants in the predialyzed patients and that other molecules with ability to reduce the ferric ions or the ABTS radicals substantially contribute to their levels.

In contrast to our (and others') results, there are also findings of lower levels of total serum antioxidants in chronically hemodialyzed patients before the hemodialysis session in comparison to healthy subjects [38, 39]. Notably, in these clinical studies were used completely different methods (in which hydroxyl radical is generated) than those evaluated in our study [40, 41].

All three antioxidant assays have shown that there was a highly significant decrease of the content of total serum antioxidants during the single hemodialysis session.

This is a common finding of many clinical studies [15, 42] which is explained with depletion of the low molecular antioxidants through the dialyzer's membrane. However, there are also some opposite findings [43].

The percentage of decrease of the levels of antioxidants during the hemodialysis session was rather high and almost identical when measured with the FRAP and TAS assays (38.6% and 35.2%, resp.) but almost three times lower when measured with the BAP assay (12.6%). This finding could possibly be explained with the fact that the coefficient of correlation between the BAP and the uric acid is the lowest in comparison to all other coefficients of correlation of the uric acid (Table 2). In addition, the lowest correlation between BAP and uric acid could mean that the BAP assay reflects better the presence of antioxidants other than the uric acid, with possible beneficial health effects. In this context, a recent study demonstrated that hemodialysis patients with higher BAP values have better survival rate [44].

Serum total antioxidants were lower in the postdialyzed patients than in the healthy subjects, which has been demonstrated by all three assays.

This difference was statistically significant for the FRAP and BAP assays only (iron reduction principle) but not for TAS assay (ABTS reduction principle). Once more this finding emphasizes the influence of different assays on the final conclusions which are withdrawn for the same sets of clinical samples.

## 5. Conclusion

Conducting this comparative analysis of some of the total (anti)oxidant assays, using the same sets of serum samples from chronically hemodialyzed patients and healthy subjects, we demonstrate that even with a tight control of all preanalytical and analytical variables (such as identical treatment of all samples, automation of most of the assays, and analysis within the same analytical series), different assays intended for measurement of the same complex “analyte,” being the total (anti)oxidants, occasionally lead to different conclusions. However, we did not get any opposite results. Discussing our findings, we highlight data from the literature about the levels of total (anti)oxidants in chronically hemodialyzed patients that are sometimes inconsistent and even opposite, which warrants careful evaluation of the results obtained from laboratory measurements.

It is noteworthy that all assays which are evaluated in this study, and many similar ones, are not specific for a particular (anti)oxidant. Besides, they have been used in many clinical studies, individually or more often as a part of panel biomarkers, and together with other clinical findings, thus contributing to get a more complete insight of the changes of the (anti)oxidant status as a result of a specific treatment or condition. Therefore, the variability of measurements should be taken into account in interpretation of data from clinical trials. This study highlights the importance of this issue in hemodialysis patients.

## Figures and Tables

**Table 1:** Coefficients of correlation between FRAP, TAS, and BAP assays, both before and after the single hemodialysis session.

		TAS		BAP	
		Before HD	After HD	Before HD	After HD
FRAP	Before HD	0.903**		0.527**	
	After HD		0.805**		0.259^ns^

TAS	Before HD			0.575**	
	After HD				0.668**

^ns^Nonsignificant.

**P < 0.01.

**Table 2:** Correlation coefficients of uric acid versus FRAP, TAS, and BAP.

Correlation coefficients	Before hemodialysis	After hemodialysis	Healthy persons
Uric acid versus FRAP	0.547**	0.430*	0.875**
Uric acid versus TAS	0.662**	0.521**	0.930**
Uric acid versus BAP	0.410*	0.385*	0.366*

*P < 0.05.

**P < 0.01.

**Table 3:** Relative contributions of individual plasma antioxidants to total FRAP and TAS values (data from literature).

	Estimated % contribution to total FRAP	Estimated % contribution to total TAS
Uric acid	60	33
Protein	10	53
Bilirubin	5	2
Ascorbic acid	15	5
*α*-Tocopherol	5	2
Others	5	5

## Abbreviations

ROM: Reactive oxygen metabolites
CARR U: Carratelli units
TOS: Total oxidant status
FRAP: Ferric reducing ability of plasma
TAS: Total antioxidant status
BAP: Biological antioxidant potential
ABTS: 2,2′-azino-bis(3-ethylbenzothiazoline-6-sulphonic acid).
